# Finite Element Modelling of a Synthetic Paediatric Spine for Biomechanical Investigation

**DOI:** 10.3390/ma16134514

**Published:** 2023-06-21

**Authors:** Nor Amalina Muhayudin, Khairul Salleh Basaruddin, Muhammad Farzik Ijaz, Ruslizam Daud

**Affiliations:** 1 Faculty of Mechanical Engineering Technology, Universiti Malaysia Perlis, Pauh Putra Campus, Arau 02600, Malaysia; noramalina@unimap.edu.my (N.A.M.); khsalleh@unimap.edu.my (K.S.B.); ruslizam@unimap.edu.my (R.D.); 2 Sports Engineering Research Centre (SERC), Universiti Malaysia Perlis, Arau 02600, Malaysia; 3 Mechanical Engineering Department, College of Engineering, King Saud University, Riyadh 11421, Saudi Arabia; 4 King Salman Center for Disability Research, Riyadh 11614, Saudi Arabia

**Keywords:** synthetic paediatric spine, finite element analysis, paediatric FE model, range of motion

## Abstract

Studies on paediatric spines commonly use human adult or immature porcine spines as specimens, because it is difficult to obtain actual paediatric specimens. There are quite obvious differences, such as geometry, size, bone morphology, and orientation of facet joint for these specimens, compared to paediatric spine. Hence, development of synthetic models that can behave similarly to actual paediatric spines, particularly in term of range of motion (ROM), could provide a significant contribution for paediatric spine research. This study aims to develop a synthetic paediatric spine using finite element modelling and evaluate the reliability of the model by comparing it with the experimental data under certain load conditions. The ROM of the paediatric spine was measured using a validated FE model at ±0.5 Nm moment in order to determine the moment required by the synthetic spine to achieve the same ROM. The results showed that the synthetic spine required two moments, ±2 Nm for lateral-bending and axial rotation, and ±3 Nm for flexion-extension, to obtain the paediatric ROM. The synthetic spine was shown to be stiffer in flexion-extension but more flexible in lateral bending than the paediatric FE model, possibly as a result of the intervertebral disc’s simplified shape and the disc’s weak bonding with the vertebrae. Nevertheless, the synthetic paediatric spine has promising potential in the future as an alternative paediatric spine model for biomechanical investigation of paediatric cases.

## 1. Introduction

Despite the fact that it was obvious that the spines of children and adults were not the same, investigation of paediatric spinal biomechanics began with modification based on an adult model. The architecture of the vertebra, the orientation of the facet joints, the ossification state of the vertebrae, and the nucleus pulposus size, were the key distinctions between the paediatric intervertebral disc and the adult disc. Because to the challenges in obtaining paediatric specimens, some studies on paediatric biomechanical examination continue to employ adult human and immature porcine spines as their specimens [1,2,3]. By creating a functional spine out of synthetic materials, this restriction can be removed. Artificial materials are now frequently utilised as an alternative in biomechanical testing, particularly when examining trabecular bone [4,5]. This initiates the process of creating a functional synthetic spine model for biomechanical investigation [6]. Wang et al. [7] compared the Sawbones model of the L3/4 motion segments with previously available information on the spines of sheep and humans. They claimed that, with the exception of axial rotation, the synthetic spine’s range of motion (ROM) was consistent with the data. The ROM of artificial spines remained within the range of human spine data even after 10,000 cycles [6]. A three-dimensional (3D) printed synthetic spine made from the L3–L5 spinal region was created by Bohl et al. [8,9]. The research revealed that, even if there were variations in ROM, the model could imitate a particular motion of the conventional ROM that was evaluated on cadavers [8]. The development of a synthetic spine that can fully match the behaviour of human cadaver is fraught with difficulties. Yet, there is also tremendous potential for a workable synthetic to revolutionise the spinal biomechanics testing field.

The other alternative to investigate paediatric mechanical behaviour is to use the finite element (FE) analysis. The reconstruction of the FE model based on the human adult spine is progressing rapidly in recent years. However, the studies of paediatric spines were mostly performed based on a scaled-down version of adult finite element models [10,11,12]. Jebaseelan et al. [13] reported that the differences in anatomy and material properties between the paediatric and adult spines are the crucial factor that impacts the outcomes. However, the recent studies on the paediatric FE model are still utilizing the comparison with adult spinal material properties due to the limited published experimental data on paediatric spines [14,15]. Using the CT scan data from a 13-month-old patient, along with the parameters of adult material properties, Phuntsok et al. [14] created a paediatric craniocervical junction. In their paediatric model, they discovered that the adult ligament characteristics with a 10% reduction indicated the ROM was within 1 standard deviation compared to the ROM of published data. Li et al. [16] generated a FE model of a 3-year-old cervical spine from CT scans by using scaled-down adult materials properties based on bone density. They used the quasi-static validations approach to validate the model against the limited published experimental data, and the results suggested that the model can be used under a certain quasi-static percentage as well. Li et al. [15], on the other hand, generated a cervical spine (C6–C7) FE model of 6-year-old and an adult from CT scans. The FE models were created using a structured multiblock method, and the study looks into the differences in C6–C7 between children and adults under various loading conditions. The study discovered that the child’s motions were significantly higher than the adult’s, with the child’s ROM being 4.57 times, 1.93 times, and 2.51 times that of the adult under the child loading condition of 0.5 Nm.

In order to study the mechanical behaviour of paediatric situations, such paediatric trauma from auto accidents, paediatric sports-related injuries, and scoliosis, a synthetic paediatric spine must be created. Hence, the authors are currently working on a project to create a working paediatric mid-thoracic (T4–T8) spinal segment model. The fabricated synthetic paediatric spine has been compared with the porcine spine on the ROM and the results suggested that the synthetic paediatric spine ROM was acceptable, as it was in good agreement with lateral-bending and axial rotation [17]. The next step is to investigate if the synthetic paediatric spine can obtain the acceptable value of ROM under certain child loading conditions using finite element analysis. Therefore, the main aims of this present study are to develop a synthetic paediatric spine FE model and to validate the FE model by comparing the ROM responses with an experimental data and paediatric model under certain loadings using finite element analysis.

## 2. Methods

### 2.1. Geometrical Modelling of Paediatric Spine

**Figure 1:**
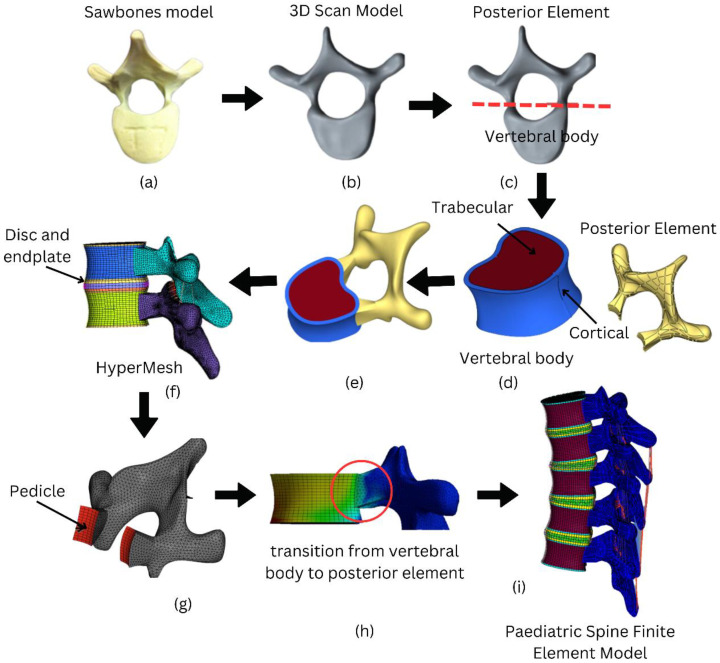
Process flow of the development of the finite element model for paediatric spine. (**a**) Sawbones model (8–9 years old), (**b**) 3D scan model, (**c**) the model is divided into vertebral body and posterior element, (**d**) reconstruction of the vertebral body to reflect trabecular and cortical bone, (**e**) model completed with trabecular and cortical bone, (**f**) created disc and endplate in between vertebra, (**g**) connected vertebral body and posterior body through pedicle, (**h**) stress distribution transition from vertebral body to posterior element, (**i**) completed paediatric spine finite element model.

The geometrical model was created using a physical model purchased from Sawbones (WA, USA) for a paediatric (8 to 9 years old) spine of T4–T8. Figure 1 depicts the overall process for developing the paediatric spine FE model. The vertebral body and the posterior element were the two components that made up a vertebra. Without making any geometrical adjustments, the posterior portion was produced based on the 3D scan model (Figure 1a,b). The Hypermesh programme was then opened after the model was imported. Using Hypermesh’s “FE-Surf” tool, a surface was created from the posterior element meshed. Next, the pedicle was then used to connect the posterior element to the cortical layer of the vertebral body. A cortical layer of 1 mm thickness was modelled in order to construct a realistic model of vertebra.

The geometrical of the vertebral body was reconstructed in PTC Creo 8 Parametric software to include cortical and trabecular bones. It is crucial to separate the cortical and trabecular bone parts because they have different mechanical properties and play different roles in the overall strength and stability of the bone. Cortical bone is denser and stiffer, providing the bone with its strength and resistance to bending and torsion. On the other hand, trabecular bone is more porous and flexible, allowing it to absorb shock and adapt to changes in loading conditions. This process can be visualized in the flow diagram as shown in Figure 1c–e.

A reference of geometry was required to develop the vertebral column, because each of the five vertebrae (T4–T8) was scanned independently. Based on the anatomical spinal curvature in a human spine CAD model as a reference, the vertebrae were stacked accordingly on top of each vertebra to complete the vertebral column (Figure 1f–i).

### 2.2. Meshing

All parts of the paediatric spine model were meshed in Hypermesh software 2021. Eight-noded brick elements were employed to mesh the trabecular, cortical, and bony and cartilaginous endplates. Mesh elements with a size of 2.0 mm were used because they passed the convergence test as the optimum element size. The intervertebral disc was created using mixed parts to mimic the biological soft tissues. Eight-noded brick elements were employed to model the annulus fibrosus base material (annulus matrix) with tension-only link elements to represent the embedded fibre. The link elements were added to the annulus matrix in a crisscross pattern at each layer. Because of the complex geometrical shape, a different approach was considered for the posterior element. Four-noded tetrahedral elements were used to preserve the geometrical shape of the posterior element. At the pedicle, the posterior element’s 4-noded tetrahedral elements were combined with the vertebral body’s 8-noded brick elements. The hybrid mesh of tetra/hexahedral elements was used to provide high accuracy and optimize the simulation time. Although there is a mixing of tetrahedral and hexahedral elements in this region, the previous work [18] showed no relevant different between the meshed model with a hexahedral only and mixture of hexa-tetrahedral elements. The reliability of the model based on connection between vertebral body and posterior element was validated by observing the stress continuity test as shown in Figure 1h. Since there is no interruption in the stress continuity, the model was accepted for further investigation.

**Figure 2:**
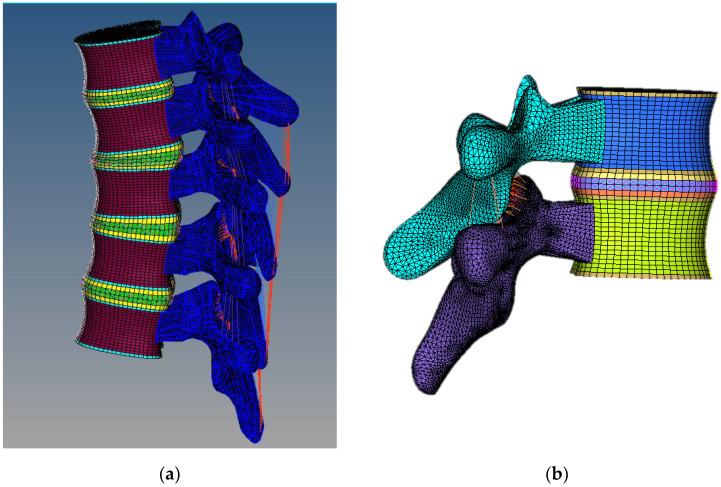
(**a**) Completed multi segment model of T4–T8. (**b**) Example of FSU model (T4–T5), which was used to perform further analysis.

Elements representing anterior longitudinal (ALL), posterior longitudinal (PLL), supraspinous (SSL), ligamentum flavum (LF), interspinous (ISL), intertransverse (TL), and facet capsulary (CL) ligaments were added to the model. Non-linear springs and link elements were used to model the spinal ligaments. To account for the pre-tension in these ligaments, the ALL, PLL, and LF were modelled as non-linear springs. The remaining ligaments were created as tension-only link elements [19,20]. Moreover, the facet joints were set as a 3D friction contact elements with a 0.01 contact stiffness factor under asymmetric contact. Figure 2 depicts the completed meshed model of multi-segment T4–T8, as well as an example of an FSU model (T4–T5). Table 1 summarises the meshing information that was applied to the finite element model.

### 2.3. Material Properties

Nonlinear materials are used in the present paediatric spine model to reveal greater accuracy in simulating the spinal movements, particularly for the soft tissue movements. As for the synthetic paediatric spine FE model, the material properties used were obtained from the material testing conducted, whereas material properties of paediatric spine were referred from the other references [13,21] as summarised in Table 2.

### 2.4. Boundary Conditions

**Figure 3:**
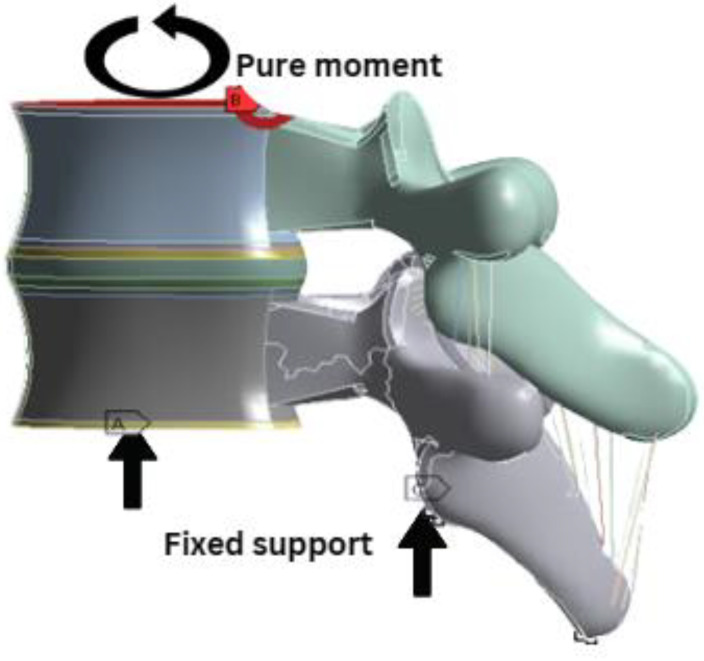
Loading and boundary conditions of all FE models. Points A and C were fixed and pure moments were applied at point B.

Pure moments of three different modes were considered and implemented to the FE model. The first loading was ±7.5 Nm, that represents adult loading. It was applied for all six degrees of freedom; flexion, extension, lateral bending, and axial rotation under all conditions as for validation of the model against the human adult spine [21,22]. Based on the data reported from previous works on similar topics, a moment of ±0.5 Nm was considered as paediatric loading, in order to obtain the correct paediatric ROM without failure [13,23]. Next, the final loading mode was applied the same as the experimental loads of ±2 Nm and ±3 Nm for all six motions, for comparison with the experimental data of previous work [17]. The bottom endplate and posterior were fixed, and the ROM (angular displacements) variable was measured as displayed in Figure 3.

### 2.5. Validation of FE Model

Nevertheless, in order to evaluate the reliability of the paediatric spine model with human tissue properties, validation of the FE model was performed [22] using a similar approach recommended by Jebaseelan et al. [13] and Kumaresan et al. [24]. Therefore, to To validate the paediatric FE model, two different boundary conditions were used. The first condition was to analyse “geometrical effect”, and the second condition was to observe the “material effect”. The results showed that there is no significant effect on geometry between the adult and paediatric FE models, but there is indeed a significant effect on the material. Despite the simplification of the vertebral body, the range of motion (ROM) was not considerably affected because the FE model was developed with a detailed anatomical of a paediatric spine consideration. Therefore, it is suggested that the FE model of the paediatric spine developed in the present can be utilized for the further investigations [22]. Hence, pure moment of ±0.5 Nm was applied to the paediatric geometry with the paediatric material in all six DOF to obtain the thoracic paediatric ROM. The ROM increased from the flexion and extension, followed by the axial rotation for all six DOF. These increases were as expected, and matched with the similar pattern reported on ROM for the adult thoracic spine by White and Panjabi [25].

### 2.6. Synthetic Spine Model Validation

For validation of synthetic paediatric FE model, comparison of the ROM between the synthetic paediatric FE model and the synthetic paediatric spine (that was obtained from the experimental data [17]) was conducted. The objective for the experimental procedure was to ensure that the fabricated synthetic model could mimic the biological specimen behaviour as tested using the Bionix Servohydraulic spine simulator [17]. The ROM curves obtained from a synthetic paediatric spine exhibited nonlinearities for all motions, because the measurements of neutral zone (NZ) and elastic zone (EZ) stiffness were less than “1”. As a result, it demonstrated that the fabricated synthetic paediatric spine behaved similarly to the biological specimen, particularly in terms of ROM. The ROM measured from the validated paediatric FE model (under 0.5 Nm of moment) was used as a reference to determine the magnitude of moment for the synthetic paediatric FE model that can produce the same ROM. Then, the same moment was used to obtain ROM for synthetic paediatric model (by experiment). The material properties of the synthetic materials were used in this analysis.

## 3. Results and Discussion

**Figure 4:**
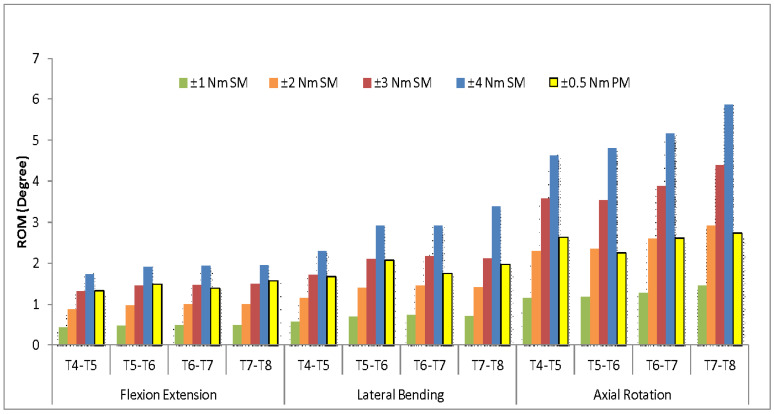
The synthetic model (SM) results from ± (1 to 4) Nm versus paediatric model (PM) (100%) at ±0.5 Nm.

Results of comparisons between the paediatric (bone) FE model under applied moments of ±0.5 Nm loads with the synthetic FE model under applied moments of between ±1 to 4 Nm loads are shown in Figure 4. Based on the graphs, the lowest average percentage differences in the flexion/extension and lateral bending movements between synthetic FE model and paediatric FE model were observed when the ±3 Nm loads were applied. In the flexion/extension movement, the average difference was measured at 3% and the difference in the lateral bending movement was measured at 9%. In contrast, the axial rotation was showing the lowest average percentage difference at the ±2 Nm loads and with a 6% difference calculated. A percentage difference of almost 50% in the axial rotation was recorded for the synthetic and paediatric models at ±3 Nm loads. Meanwhile, a percentage difference of approximately 30% in the flexion/extension and lateral bending movements was found for the synthetic FE model at ±2 Nm loads.

**Figure 5:**
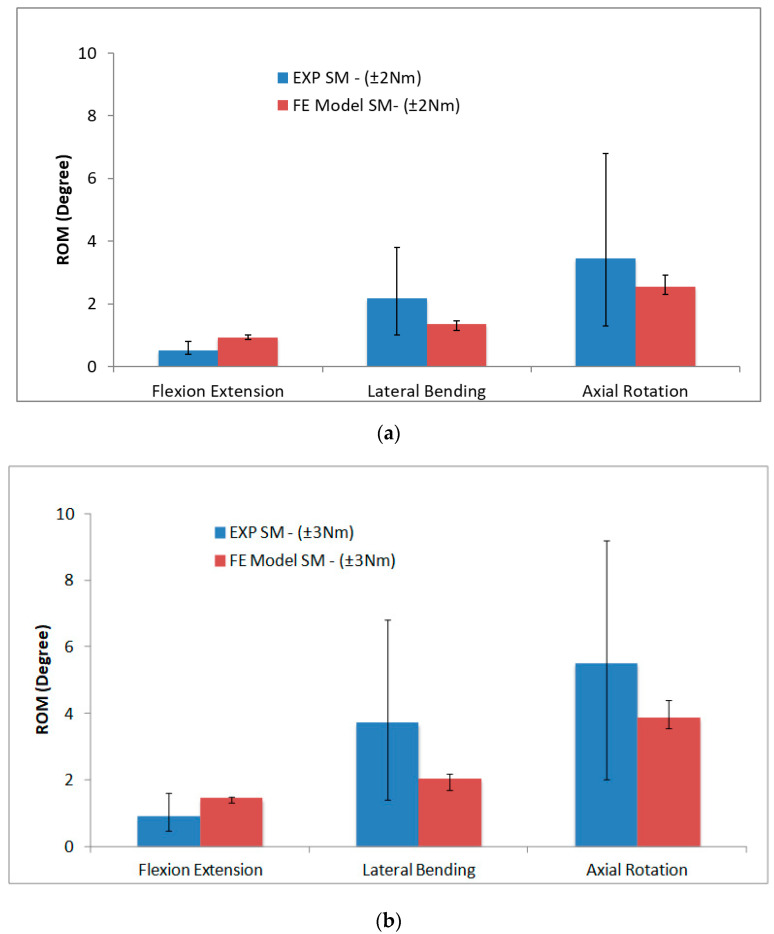

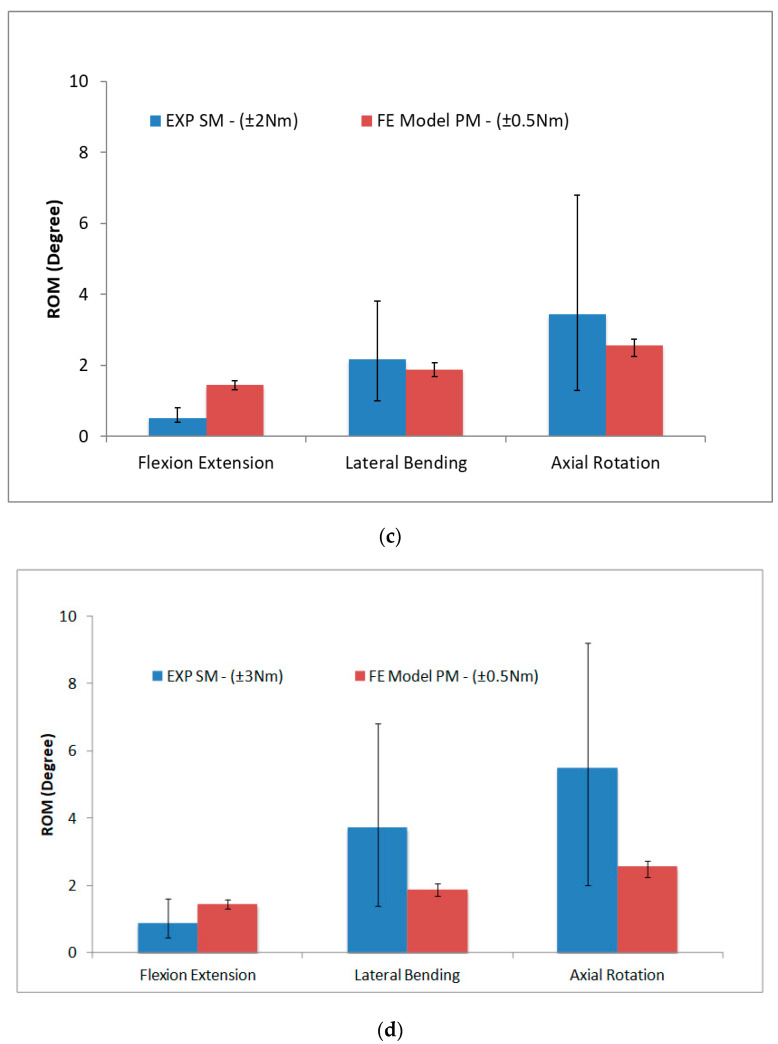
ROM of experimental and FE model of synthetic paediatric spine under: (**a**) ±2 Nm moment, (**b**) ±3 Nm moment, (**c**) ±2 Nm moment of experimental vs. ±0.5 Nm moment of paediatric (bone) FE model and (**d**) ±3 Nm moment of experimental vs. ±0.5 Nm paediatric (bone) FE model.

Further analysis of the synthetic FE model was carried out to compare the ROM against the experimental data [17]. Based on the average percentage differences, the synthetic FE model ROM at ±2 Nm loads for the flexion/extension and lateral bending movements were the closest to the experimental result of paediatric ROM. For the axial rotation movement, the ±3 Nm loads of the synthetic FE model had the lowest percentage of ROM difference, as compared with the paediatric FE model. Therefore, both of the moments were subsequently compared with the experimental data as depicted in Figure 5. The average ROM of the synthetic paediatric spine for both the moments and for all six DOF of experimental data were higher than the values reported in the FE model, apart from the flexion/extension. The average differences for both moments varied from 30% to 60%, while the average T4–T8 ROM of synthetic paediatric FE model were within the data set range, according to the experimental data. As presented in Figure 5, the ROM from experimental data increased significantly (by 50%) from ±2 Nm to ±3 Nm loads, which suggested that the synthetic paediatric spine was more flexible after the ±2 Nm loads.

**Figure 6:**
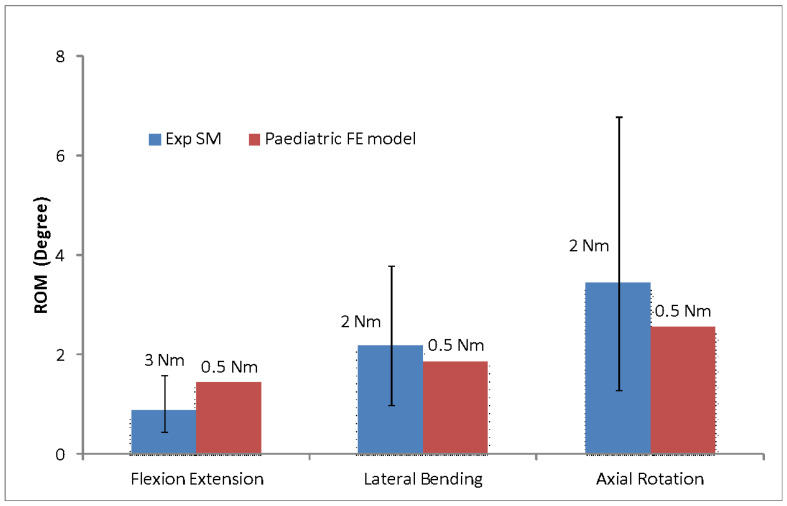
The experimental data of the synthetic paediatric model that matched the ROM of paediatric FE model at ±0.5 Nm.

In order to obtain the paediatric ROM, the experimental data for both the moments (i.e., ±3 Nm and ±2 Nm load) were compared with the paediatric FE model at ±0.5 Nm, as presented in Figure 6. A similar pattern for the ±2 Nm loads was observed in this comparison data as well, with the smaller percentage differences occurring particularly in the lateral bending (15%) and axial rotation (30%) movements. In contrast, the flexion/extension was the closest under the ±3 Nm loads, with a 48% difference, as compared to the 96% difference at ±2 Nm loads. Again, this value was within the set of data range of the synthetic paediatric spine from the experimental data. These comparisons suggested that to obtain the paediatric ROM, two different moments are required for each direction in ROM.

The comparison of the synthetic paediatric FE and paediatric (bone) FE models showed good lateral bending and flexion/extension agreements at ±3 Nm, while good axial rotation agreement was shown at ±2 Nm. These similar results were expected for the experimental data of the synthetic paediatric spine. The ROM in terms of axial rotation and flexion/extension observed in the synthetic paediatric FE model was comparable with the experimental data. However, the ROM in lateral bending obtained in the experiment was closer to the ±2 Nm loads, suggesting that it was more flexible in the experimental model than the FE model. The lateral bending and axial rotation of the paediatric FE model were closer to the synthetic paediatric spine at ±2 Nm loads, with 15% and 29% differences measured, respectively. Since the ROM in flexion/extension was found stiffest, the closest motion was at ±3 Nm moment with 48% differences, but the value was still falling within the range of experimental data. The final comparison is summarised in Figure 6. This movement depends mostly on the nonlinearity behaviour of the intervertebral disc. Therefore, it is vital to observe the stress distributions that occur in the disc during various loading conditions.

Further qualitative analysis was conducted to observe the significance of fibres within the annulus fibrosus matrix situated inside the intervertebral disc, using the FE model as the synthetic disc in this research, which was fabricated without the fibres. This analysis was carried out since the viscoelasticity of the intervertebral disc had a significant effect on lateral bending and flexion extension. The stress distribution in the intervertebral disc during lateral bending between the human disc was observed and studied based on the embedded fibres in the synthetic disc by employing the synthetic spine model. The maximum principal stress was used to compare the results, to show the maximum strength value at the tension elastic limit. A similar response was observed in this study in comparison with Ryan et al. [26], who had previously investigated and established a correlation between stress distributions in the intervertebral disc of the porcine lumbar spine. The lower stress can be observed on the side that was compressed, while the higher stress was on the side that was extended when the lateral bending motion was applied to the model. The stress value was significantly lower in the human disc because the tension forces were carried by the fibres, and not by the annulus fibrosus matrix, while in the synthetic disc, the tension forces were carried by the annulus fibrosus itself.

Although the stress values in the human disc were significantly lower than the synthetic disc, both of these discs exhibited the same ‘tension’ behaviour that mainly occurred on the extension movement side. One of the potential causes for this difference may be due to the geometric design of the intervertebral disc used in the synthetic paediatric spine to replicate the human disc. Since the shape of the intervertebral disc was simplified for easy manufacturing, some gaps in between of the vertebral bodies were also created. In contrast, the intervertebral disc in the FE model was designed based on the gap between the vertebral bodies. The gap between the vertebral disc in the synthetic paediatric disc was filled with silicone plastic. The gap may have allowed more movements, thus contributing to flexibility as it did not hold the vertebral body together. This may have been a factor that causes the significant difference in lateral bending and, to a lesser extent, the stiffness motions found for the flexion/extension. However, the synthetic disc was fabricated purely to provide movement for the synthetic paediatric spine; thus, the movement mechanism was focused. In this research, the synthetic paediatric disc was in good agreement with the human paediatric disc, based on the findings obtained from comparison of the two FE models. Accordingly, these findings suggested that the synthetic paediatric disc fabricated in the synthetic paediatric spine was acceptable. Here, we also envisage that the beta titanium alloys having advantageous mechanical properties that could serve promising potential for the fabrication of paediatric spines and other biomedical devices [27,28,29,30].

## 4. Conclusions

The main aim of the present this study was to develop a reliable finite element model of a synthetic paediatric spine that can behave similar to the ROM of the actual paediatric spine under certain loading conditions. The results showed that the flexion/extension and lateral bending of the paediatric spine were close to synthetic paediatric FE model under ±3 Nm moment while the axial rotation was at ±2 Nm. Comparing the ROM of the synthetic paediatric FE model at ±2 and ±3 Nm moment with experimental data, it showed that the experimental data was more flexible than the FE model, except for flexion/extension, but the values were still within range. Interestingly, the results also showed that the lateral bending and axial rotation were closed with the ±2 Nm value, while the flexion/extension value was at ±3 Nm. To summarise, the analyses indicated that the synthetic paediatric spine fabricated in this research required two moments (i.e., ±2 and ±3 Nm) to obtain the ROM of paediatric spine. These differences are potentially due to the limitations of the synthetic paediatric spine model, specifically the designs of the disc and materials used to replicate the facet joint. The performances of the physical model showed a smooth lateral bending and axial rotation, but overly stiff flexion/extension. However, with further adjustment and tuning, the improved synthetic paediatric spine can potentially benefit the researcher to accurately investigate more complicated paediatric cases that require paediatric cadaver testing. In addition, it minimised the problems, such as the large variation between specimens, which occurred with cadaveric testing, and it has the flexibility to be moulded into complicated shapes, for example to mimic the spinal shape of patients suffering from scoliosis. Development of a working synthetic paediatric spine could potentially be used for surgical intervention judgements in future, especially for spine deformity cases in paediatrics.

## Figures and Tables

**Table 1:** Meshing specifications for each component in the FE model.

Component	Element	
	Type	Number
Bony endplate	8-noded brick	594
Cortical shell	8-noded brick	1024
Trabecular bone	8-noded brick	8480
Posterior element	4-noded tetra	24,960–30,559
Cartilage endplate	8-noded brick	594
Nucleus Pulposus	8-noded brick	676
Annulus Fibrosus matrix	8-noded brick	512
Disc Fibres	2-noded truss	336
ALL	Nonlinear spring	200
PLL	Nonlinear spring	120
ISL	2-noded truss	5
SSL	2-noded truss	5
TL	2-noded truss	4
CL	2-noded truss	30
LF	Nonlinear spring	6

**Table 2:** Material properties of synthetic materials and paediatric spine.

	Synthetic Materials		Paediatric Spine	
Component	Young’s Modulus (MPa)	Poisson’s Ratio	Young’s Modulus (MPa)	Poisson’s Ratio
**Bone**				
Cortical bone	3036	0.25	E_xx_ = 645.7 E_yy_ = 645.7 E_zz_ = 1257	ν_xy_ = 0.484 ν_xz_ = 0.203 ν_xz_ = 0.203
Trabecular bone	90	0.2	E_xx_ = 105.3 E_yy_ = 105.3 E_zz_ = 150	ν_xy_ = 0.450 ν_xz_ = 0.315 ν_xz_ = 0.315
Cartilage Endplate/Growth Plate	7.5	0.45	23.8	0.4
Posterior bone	90	0.2	200	0.25
**Intervertebral disc**				
Nucleus pulposus (fluid)	1.6	0.4999	0.2	0.4999
Annulus Fibrosus matrix	15.63	0.45	Hyperelastic, Mooney Rivlin, C_1_-0.12, C_2_-0.03	
**Spinal Ligaments**				
ALL	Force-deflection curve of synthetic ligaments.		90% of adult ligament stiffness value	
PLL				
LF				
TL	0.62	0.3	52.83	0.3
ISL			10.44	0.3
SSL			13.5	0.3
CL			29.61	0.3

## Data Availability

The data obtained in this study are available from the corresponding author upon request.

## References

[1] Shiba K., Taneichi H., Namikawa T., Inami S., Takeuchi D., Nohara Y. (2017). Osseointegration improves bone–implant interface of pedicle screws in the growing spine: A biomechanical and histological study using an in vivo immature porcine model. Eur. Spine J..

[2] Lavelle W.F., Moldavsky M., Cai Y., Ordway N.R., Bucklen B.S. (2016). An initial biomechanical investigation of fusionless anterior tether constructs for controlled scoliosis correction. Spine J..

[3] Fekete T.F., Kleinstück F.S., Mannion A.F., Kendik Z.S., Jeszenszky D.J. (2011). Prospective study of the effect of pedicle screw placement on development of the immature vertebra in an in vivo porcine model. Eur. Spine J..

[4] Nowak B. (2019). Experimental study on the loosening of pedicle screws implanted to synthetic bone vertebra models and under non-pull-out mechanical loads. J. Mech. Behav. Biomed. Mater..

[5] González S.G., Jiménez J.F.V., Bastida G.C., Vlad M.D., López J.L., Aguado E.F. (2020). Synthetic open cell foams versus a healthy human vertebra: Anisotropy, fluid flow and μ-CT structural studies. Mater. Sci. Eng. C.

[6] Muhayudin N.A., Basaruddin K.S., Yazid H., Salleh A.F. (2021). Development of synthetic spine for biomechanical research: An overview. J. Phys. Conf. Ser..

[7] Wang T., Ball J.R., Pelletier M.H., Walsh W.R. (2014). Biomechanical evaluation of a biomimetic spinal construct. J. Exp. Orthop..

[8] Bohl M.A., Mooney M.A., Repp G.J., Nakaji P., Chang S.W., Turner J.D., Kakarla U.K. (2018). The barrow biomimetic spine. Spine.

[9] Bohl M.A., McBryan S., Newcomb A.G.U.S., Lehrman J.N., Kelly B.P., Nakaji P., Chang S.W., Uribe J.S., Turner J.D., Kakarla U.K. (2019). Range of Motion Testing of a Novel 3D-Printed Synthetic Spine Model. Glob. Spine J..

[10] Kumaresan S., Yoganandan N., Pintar F.A., Maiman D.J. (2000). Biomechanical study of pediatric human cervical spine: A finite element approach. J. Biomech. Eng..

[11] Kumaresan S., Yoganandan N., Pintar F.A. (1999). Finite element analysis of the cervical spine: A material property sensitivity study. Clin. Biomech..

[12] Meijer G.J.M. (2011). Development of A Non-Fusion Scoliosis Correction Device. Ph.D. Thesis.

[13] Jebaseelan D.D., Jebaraj C., Yoganandan N., Rajasekaran S. (2010). Validation efforts and flexibilities of an eight-year-old human juvenile lumbar spine using a three-dimensional finite element model. Med. Biol. Eng. Comput..

[14] Phuntsok R., Mazur M.D., Ellis B.J., Ravindra V.M., Brockmeyer D.L. (2016). Development and initial evaluation of a finite element model of the pediatric craniocervical junction. J. Neurosurg. Pediatr..

[15] Li Z., Song G., Su Z., Wang G. (2020). Development, validation, and application of ligamentous cervical spinal segment C6–C7 of a six-year-old child and an adult. Comput. Methods Programs Biomed..

[16] Li N., Wei W., Wu S., Du X., Liu Y., Rong P. (2019). Development of a 3-y-old Pediatric Cervical Spine Finite Element Model. IOP Conf. Ser. Mater. Sci. Eng..

[17] Muhayudin N.A., Basaruddin K.S., Daud R., McEvoy F., Tansey (2021). Experimental Analysis of Fabricated Synthetic Midthoracic Paediatric Spine as Compared to the Porcine Spine Based on Range of Motion (ROM). Appl. Bionics Biomech..

[18] Ruggiero A., D’Amato R., Affatato S. (2019). Comparison of Meshing Strategies in THR Finite Element Modelling. Materials.

[19] Sim O., Ryu D., Lee J., Lee C. (2022). Stress distribution on spinal cord according to type of laminectomy for large focal cervical ossification of posterior longitudinal ligament based on finite element method. Bioengineering.

[20] Xu H., Wu J., Xie H., Wen W., Xu H., Du J., Miao J. (2022). Biomechanical behaviour of tension-band-reconstruction titanium plate in open-door laminoplasty: A study based on finite element analysis. BMC Musculoskelet. Disord..

[21] Panjabi M.M., Brand R.A., White A.A. (1976). Mechanical properties of the human thoracic spine as shown by three-dimensional load-displacement curves. J. Bone Jt. Surg..

[22] Muhayudin N.A., Basaruddin K.S., McEvoy F., Tansey A. (2021). Development and evaluation of a paediatric mid-thoracic spine finite element model. AIP Conf. Proc..

[23] Ouyang J., Zhu Q., Zhao W., Xu Y., Chen W., Zhong S. (2005). Biomechanical assessment of the pediatric cervical spine under bending and tensile loading. Spine.

[24] Kumaresan S., Yoganandan N., Pintar F.A. (1998). Finite element modeling approaches of human cervical spine facet joint capsule. J. Biomech..

[25] White A., Panjabi M. (1990). Clinical Biomechanics of the Spine.

[26] Ryan G., Pandit A., Apatsidis D. (2008). Stress distribution in the intervertebral disc correlates with strength distribution in subdiscal trabecular bone in the porcine lumbar spine. Clin. Biomech..

[27] Ijaz M.F., Kim H.Y., Hosoda H., Miyazaki S. (2015). Superelastic properties of biomedical (Ti-Zr)-Mo-Sn alloys. Mater. Sci. Eng. C Mater. Biol. Appl..

[28] Ijaz M.F., Laille D., Heraud L., Gordin D., Castany P., Gloriant T. (2016). Design of novel superelastic Ti-23Hf-3Mo-4Sn biomedical alloy combining low modulus, high strength and large recovery strain. Mater. Lett..

[29] Ijaz M.F., Alharbi H.F., Bahri Y.A., Sherif E.S.M. (2022). Alloy design and fabrication of duplex titanium-based alloys by spark plasma sintering for biomedical implant applications. Materials.

[30] Héraud L., Castany P., Ijaz M.F., Gordin D.M., Gloriant T. (2023). Large-strain functional fatigue properties of superelastic metastable β titanium and NiTi alloys: A comparative study. J. Alloys Compd..

